# One-step balloon-assisted direct peroral cholangioscopy prior to placing the anti-reflux self-expandable metal stent

**DOI:** 10.1055/a-2085-0615

**Published:** 2023-05-26

**Authors:** Seiji Fujigaki, Tsuyoshi Sanuki, Akira Shirohata, Ryusuke Ariyoshi, Katsuhide Tanaka, Teruhisa Morikawa, Yoshikazu Kinoshita

**Affiliations:** Department of Gastroenterology, Hyogo prefectural Harima-Himeji General Medical Center, Hyogo, Japan

Endoscopic biliary drainage with a self-expandable metal stent (SEMS) is the standard treatment for malignant biliary obstructions. Duodenobiliary reflux, which is an unavoidable concern after SEMS placement, results in stent dysfunction 1 . The usefulness of a duckbill-type anti-reflux self-expandable metal stent (D-ARMS) for recurrent biliary obstruction (RBO) has been reported 2 .

A 78-year-old woman who had undergone SEMS placement for ampullary carcinoma was admitted to our hospital with acute cholangitis caused by RBO ( Fig. 1 ). The placement of the D-ARMS within the lumen of the SEMS was attempted to prevent duodenobiliary reflux. To avoid early stent dysfunction due to food impaction inside the anti-reflux valve, food and sludge should not be in the bile duct before placement of the D-ARMS. Therefore, direct peroral cholangioscopy (DPOCS) using an ultraslim gastroscope (GIF-1200N; Olympus, Tokyo, Japan) was used to confirm bile duct clearance ( Video 1 ). This scope has a large (2.2 mm) working channel; therefore, a balloon catheter (B5-2Q, Olympus) with a 0.018-inch guidewire (Fielder 18, Olympus) can be used as the anchoring device. Initially, the endoscope was advanced toward the inferior duodenal angulus. The scope was then turned, and the ampulla was observed in the retroflex position. The balloon catheter was placed deep into the left intrahepatic bile duct, following the guidewire, and the balloon was inflated to anchor the endoscope. By pushing the scope and pulling the balloon, the scope was easily advanced toward the proximal bile duct ( Fig. 2 ). Next to the DPOCS procedure, the scope was exchanged with a duodenoscope, and the D-ARMS was placed ( Fig. 3 ). No adverse events were observed, and no stent dysfunction occurred after treatment.

**Fig. 1:**
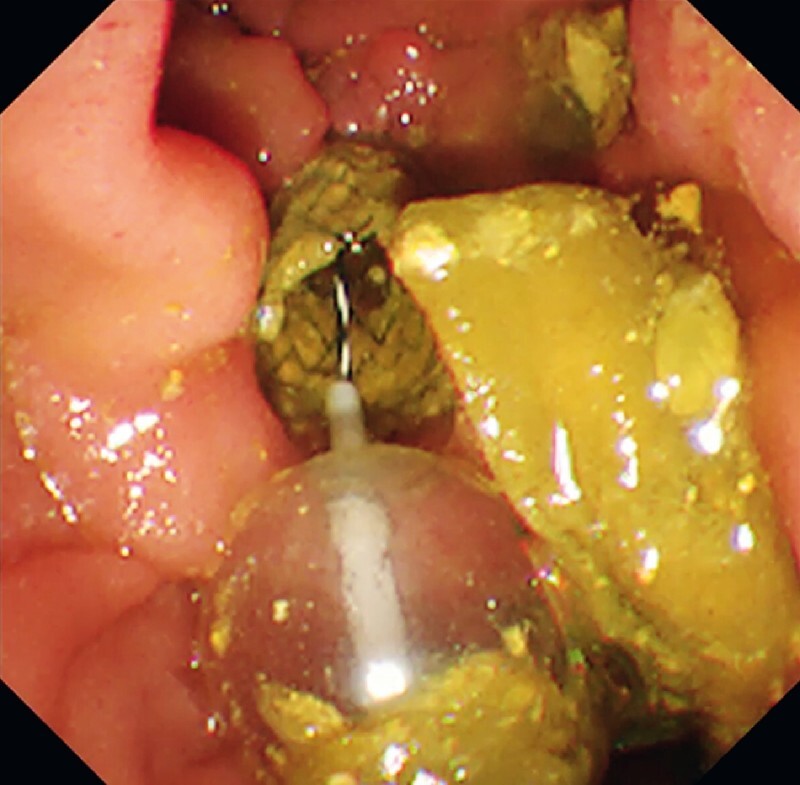
A large amount of sludge and food residue was extracted with a balloon sweep of the bile duct.

**Video 1** One-step balloon-assisted direct peroral cholangioscopy procedure and placement of a duckbill-type anti-reflux self-expandable metal stent.


**Fig. 2:**
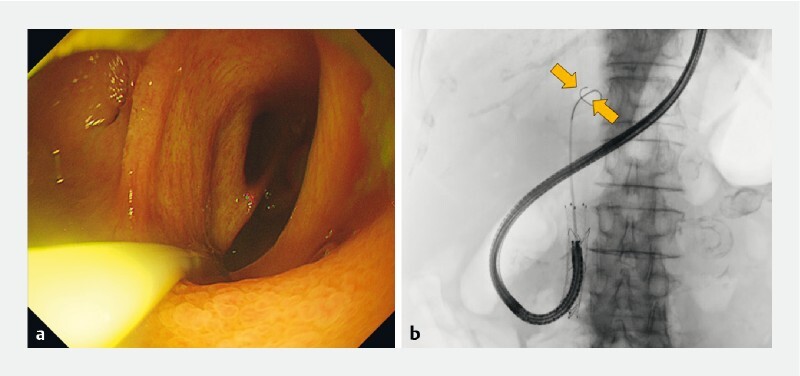
One-step balloon-assisted direct peroral cholangioscopy. **a** Endoscopic image showing clearance of the bile duct. **b** Fluoroscopic image showing the scope position and the inflated balloon (arrow).

**Fig. 3:**
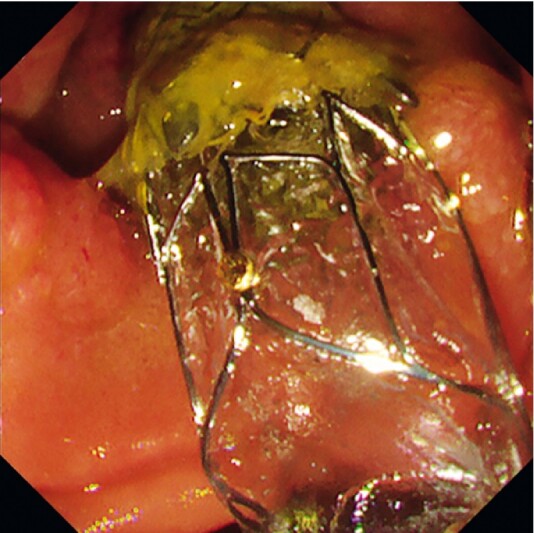
Endoscopic image showing the duckbill-type anti-reflux self-expandable metal stent placed across the papilla.

Endoscopy_UCTN_Code_TTT_1AR_2AH

## Notes

Endoscopy E-Videos

https://eref.thieme.de/e-videos
*E-Videos* is an open access online section of the journal *Endoscopy* , reporting on interesting cases and new techniques in gastroenterological endoscopy. All papers include a high-quality video and are published with a Creative Commons CC-BY license. Endoscopy E-Videos qualify for HINARI discounts and waivers and eligibility is automatically checked during the submission process. We grant 100% waivers to articles whose corresponding authors are based in Group A countries and 50% waivers to those who are based in Group B countries as classified by Research4Life (see: https://www.research4life.org/access/eligibility/ ). This section has its own submission website at https://mc.manuscriptcentral.com/e-videos
